# The right vertical infra-axillary incision for mitral valve replacement

**DOI:** 10.1186/1749-8090-5-104

**Published:** 2010-11-07

**Authors:** Qing-guo Li, Qiang Wang, Dong-jin Wang

**Affiliations:** 1 Department of Cardiothoracic Surgery, the Affiliated Drum Tower Hospital of Nanjing University Medical School, Nanjing, Peoples Republic of China

## Abstract

**Background:**

As the physiologic results of valve surgery have improved dramatically in recent years, the cosmetic effect of the procedure gains increased attention, and various alternatives to the standard median sternotomy have been developed for mitral valve surgery. We report a new minimally invasive and cosmetic approach for mitral valve replacement.

**Methods:**

From December 2003 to December 2009, the right vertical infra-axillary incision (RVIAI) was employed to perform mitral valve replacement in 256 patients. 62.9% patients had replaced mechanical valve, others were bioprosthetic valve, at the same time 28.1% patients received tricuspid valvuloplasty.

**Results:**

There were one hospital death in this series due to multiple organ failure, one reoperation for bleeding and one incision infection. Mean follow-up duration was 42.8 months (range, 3 to 72), and follow-up rate was 94%. There were no paravalvular leaks or late death during the follow up.

**Conclusions:**

The RVIAI can be performed with favorable cosmetic and clinical results. It provides a good alternative to standard median sternotomy for MVR in selected patients.

## Background

As the physiologic results of valve surgery have improved dramatically in recent years, perhaps only nonaesthetic scarring is all that remains to be improved regarding mitral valve surgery and its follow-up. Therefore, the cosmetic effect of the procedure gains increased attention, and various alternatives with favorable clinical results to the standard median sternotomy have been developed for mitral valve surgery that can avoid the characteristic unsightly, long midline scar [1-7].

Right vertical infra-axillary incision (RVIAI) has been used for repair of atrial septal defect, partial atrioventricular septal defect and ventricular septal defect[8-10], and has proved to be a safe and cosmetic alternative to median sternotomy by same authors in different period. With the accumulated experience, application of the incision had been consciously extended to mitral valve replacement for selected 256 patients.

## Methods

### Patient population

From December 2003 to December 2009, the right vertical infra-axillary incision (RVIAI) was employed to perform mitral valve replacement in 256 patients (Demographic data and diagnoses of patients listed in Table 1). Patients who required aortic valve surgery according to preoperative echocardiography or with body mass index (BMI) greater than 30 kg/m^2 ^were not recommended for RVIAI. All patients underwent MVR with or without tricuspid valvuloplasty by the same surgical team.

**Table 1:** Demographic data and diagnoses of patients

Category	Data
Age (range)	38.6 ± 8.2 (21~56)
Female	170 (66.4%)
*New York Heart Association class*	
Class I	46 (18%)
Class II	171 (66.8%)
Class III	38 (14.8%)
Class V	1 (0.4%)
*Etiology*	
Rheumatic valve disease	224 (87.5%)
Degeneration disease	32 (12.5%)
Atrial fibrillation	66 (25.6%)
Ejection fraction (range)	0.52 ± 0.11 (0.40-0.73)

### Operative technique

**Figure 1:**
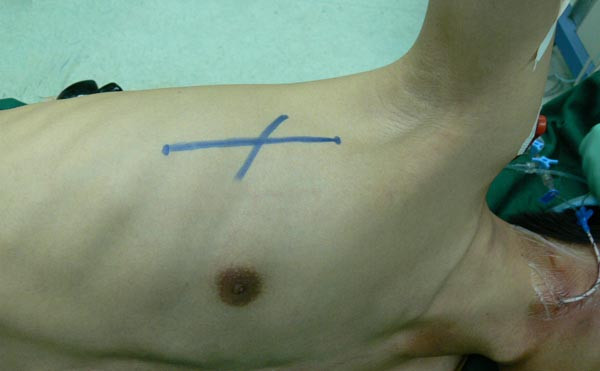
**Demonstration of position with patient and length of the incision**.

The patient is positioned with the chest in an 60~90° left lateral position and the pelvis in a corresponding 90° position. The right arm is put over the head with shoulder-joint abducted approximately 120 degrees and elbow joint in right angle position. The skin incision began at the second intercostal space along the right midaxillary line extending to the fifth intercostals space along the preaxillary line, which form a right vertical infra-axillary incision (Figure 1). The length of the incision is approximately 7 to 10 cm but varied depending upon patients' physical characteristics such as body height and weight.

The thoracic cavity is entered through the fourth intercostals space, but in asthenic type patients through the third intercostals space and in pyknic type patients through the fifth. Two retractors are used to exposure thoracic cavity. The lung is retracted posteriorly using wet sponges to expose the pericardium. The pericardium is opened 2 cm anterior to the phrenic nerve, superiorly to the pericardial reflection and inferiorly to the diaphragm, to provide enough exposure of the ascending aorta and inferior vena cava. Pericardial traction stay sutures are placed at the superior, middle, and inferior aspects of the incision. Through pericardial traction the heart can be raised 3~5 cm to skin incision. The superior pericardial stay stitches are placed on partial pleura of ribs to elevate the aorta into the operative field. Another skin incision length about 2 cm is placed at the seventh intercostal space along the right midaxillary line which place the inferior vena cava cannula in operation, and as the right pleural drain passageway after operation.

**Figure 2:**
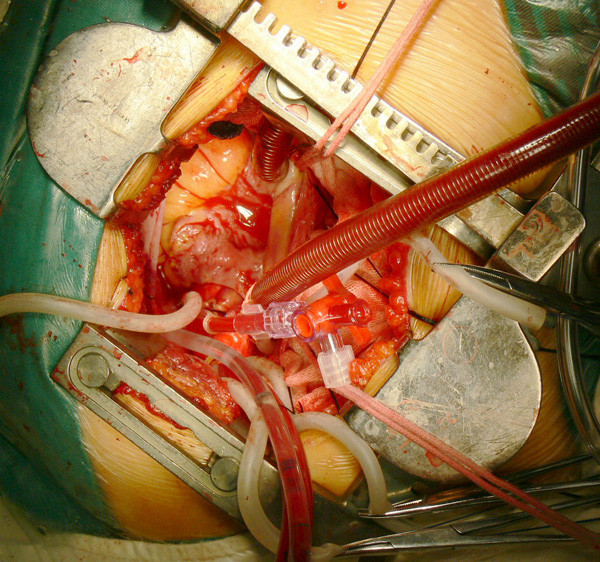
**Demonstration that all cannulations were sit down, cardiopulmonary bypass and cardioplegia were applied by the usual technique**.

Standard purse string sutures are placed on the lateral aspect of the ascending aorta and at the right atrial-superior vena caval and right atrial-inferior vena caval junctions. Tapes are passed around the vena cava in standard fashion. After heparin sodium administration, the aorta is cannulated with the help of two long vascular clamps. In common straight tip aortic cannula was used in adult. One clamp draws the cannulation site down, and the other holds the top of the aortic cannula to push it in place. With this technique, aortic cannulation in our series was accomplished without difficulty in any patient. Then the superior vena cava and inferior vena cava are cannulated. Cardiopulmonary bypass with mild hypothermia (32°C) is instituted. An aortic needle vent is connected to continuous suction, and the caval tapes are snared(Figure 2).

**Figure 3:**
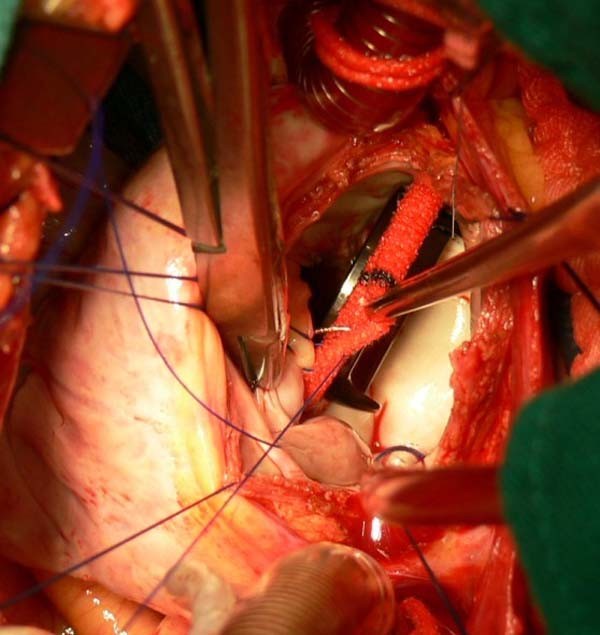
**Demonstration that the mitral valve operation is performed through the interatrial groove incision and running suture in mechanical valves replacement is usually used with 2-0 prolene line**.

The mitral valve operation is performed through the interatrial groove incision which could provide good exposure by four traction stitches at superior, inferior, anterior and posterior aspects of the incision, and the right atrium is opened when tricuspid valvuloplasty is needed. If the interatrial groove incision is narrow to result in difficult exposure, the way via the right atriotomy and the septum should be used in a trifle of cases. Running suture in mechanical valves replacement is usually used with 2-0 prolene line(Figure 3). When with difficult exposure, one or two wet sponges should be placed in the pericardial cavity beneath the heart to raise mitral valve position to provide acceptable vision, or total interrupted suture could be used, the traction form first sutures at posterior mitral valve ring could provide better exposure for near stitches. In bioprosthetic valve replacement total interrupted suture should be used, because running suture may injure bioprosthetic valve leaflet in so deep mitral position and the high struts of tissue valves also make running suture become more difficulty. The heart function and prosthesis function are monitored by transesophageal echocardiography. Pacing wires are routinely set on the ventricle of the heart in case of emergency need. After the completion of MVR, the pericardium and the thoracotomy are closed in the common fashion with a single right pleural drain at the seventh intercostal space incision. The distal end of chest tube was placed in the pericardial space through the pericardial incision to prevent postoperative cardiac temponade.

## Results

There were no patient need to extend the inciseon, or conversion to another approach in this series. Intraoperative and postoperative results listed in Table 2. There were one hospital death in this series due to multiple organ failure, one reoperation for bleeding and one incision infection. Mean follow-up duration was 42.8 months (range, 3 to 72), and follow-up rate was 94%. There were no paravalvular leaks or late death during the follow up. One case of cerebral hemorrhage happened 6 months after surgery and no anticoagulation-associated complications.

**Table 2:** Intraoperative and postoperative results

Category	Data
Mechanical valve	161 (62.9%)
Bioprosthetic valve	95 (37.1%)
Tricuspid valvuloplasty	72 (28.1%)
Aortic clamp time (min)	70.2 ± 18.2
Time to establish cardiopulmonary by pass (min)	42.4 ± 9.6
Cardiopulmonary bypass time (min)	105.3 ± 16.2
Total operation time (min)	202.7 ± 17.2
Incision length (cm)	10.3 ± 2.4
Mechanical ventilation time (hours)	5.2 ± 1.4
Drainage (mL)	237 ± 32
Hospital stay (days)	8.6 ± 1.3

## Discussion

Our approach is here compared with several newer techniques for minimally invasive heart surgery to demonstrate the reason we introduced RVIAI in our center. The internal mammary artery is prone to be damaged and cannulation of the femoral artery is usually required for parasternal incision, as reported by Navia and Cosgrove [11] and Cosgrove and Sabik [12]. The right anterolateral thoracotomy can avoid the use of femoral artery cannulation but sometimes results in thorax deformity and injury of the mammary gland of young female patients [13]. Specific instruments, additional expenses in the operating room, and the risk of aortic dissection deriving from cannulation of the femoral artery are shortcomings of port access, which had been considered to be a safe and promising technique for mitral valve surgery [14,15]. Partial sternotomy can be performed with acceptable clinical results, avoiding femoral artery and vein cannulation, but a midline scar is not popular, especially with young female patients [16].

**Figure 4:**
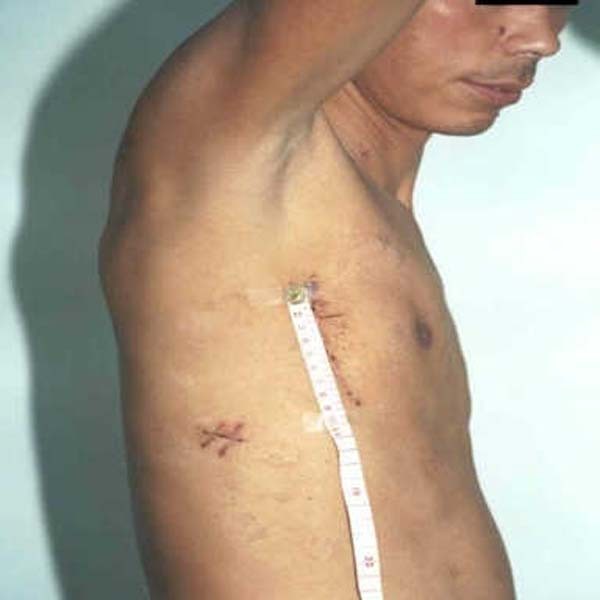
**Result of sikn incision after mitral valve replacement through right vertiacal infra-axillary incision (2 weeks after surgery)**.

The skin incision of RVIAI (Figure 4) locates posterior and superior to the right anterolateral thoracotomy and the right axillary incision described by Hitendu et al.[17], therefore it can provide enough exposure of the ascending aorta. Aortic cannulation can be completed in the incision and avoid use of femoral artery cannulation. Once the cardiopulmonary bypass is established smoothly, RVIAI increased neither aortic-clamp time nor total operating time. Because of the access can provide the vertical plane of vision to interatrial groove and AV valves, it could provide better exposure of mitral valve than other incisions.

Aortic cannulation is one of the most critical steps in the operation. In common straight tip aortic cannula was used in adult, curved tip cannula was sometimes used in children congenital heart surgery. Because the distance of the incision to aorta is farer than other access so it is difficult to use curved tip aortic cannula in deep thoracic cavity. It also is overriding shortcoming of the access that opreation field exposure is relative difficult in patients with high body mass index (BMI). Several methods could be used to raise the heart and mitral valve position, such as through pericardial traction stay suture and placement of wet sponges in the pericardial cavity beneath the heart. But wider bony thorax patients may remain difficult exposure, so patients with BMI greater than 30 kg/m2 are not recommended for RVIAI. Because increasing BMI makes aortic cannulation and operative procedure more demanding. At the same time suffered from right pleurisy or pericarditis, re-operative mitral valve procedures and old patients accompanying ascending aorta calcification are relative contraindications for RVIAI.

## Conclusions

The RVIAI can be performed with favorable cosmetic and clinical results. It provides a good alternative to standard median sternotomy for MVR in selected patients.

## Consent

Written informed consent was obtained from the patient for publication of the accompanying images. A copy of the written consent is available for review by the Editor-in-Chief of this journal.

## Competing interests

The authors declare that they have no competing interests.

## Authors' contributions

QL and DW designed the research and performed the majority of the research; DW coordinated the study in addition to providing financial support for this work; QL and QW analyzed the available data and wrote the manuscript. All authors read and approved the final manuscript.
